# Frailty Intervention Trial iN End-Stage patientS on haemodialysis (FITNESS): study protocol for a randomised controlled trial

**DOI:** 10.1186/s13063-018-2842-x

**Published:** 2018-08-24

**Authors:** Benjamin M. Anderson, Mary Dutton, Edward Day, Thomas A. Jackson, Charles J. Ferro, Adnan Sharif

**Affiliations:** 1 Department of Nephrology, University Hospitals Birmingham NHS Foundation Trust, Birmingham, UK; 2 National Addiction Centre, Institute of Psychiatry, Psychology and Neuroscience, King’s College London, London, UK; 3 Department of Geriatrics, University Hospitals Birmingham NHS Foundation Trust, Birmingham, UK; 4 Institute of Inflammation and Ageing, University of Birmingham, Birmingham, UK; 5 Institute of Immunology and Immunotherapy, University of Birmingham, Birmingham, UK

## Abstract

**Background:**

Frailty is a state of low physiological reserve and multi-systemic dysregulation that leads to susceptibility to external stressors; it is associated with adverse outcomes. North American data suggest that haemodialysis recipients are more likely to be frail than the general population, although data on UK cohorts are lacking. Furthermore, with a multitude of assessment tools, it is difficult for the clinician to ascertain which is most suitable for this population. The FITNESS Study aims to measure the prevalence and outcomes associated with frailty in a large UK haemodialysis cohort to determine the optimum frailty tool as defined by predictive value for mortality/hospitalisation and to conduct a feasibility study exploring a multi-disciplinary clinical intervention to improve frailty among haemodialysis recipients.

**Methods/design:**

The study will follow a cohort multiple randomised controlled trial design; the initial cohort study will identify participants to be invited into a subsequent open-label randomised controlled trial. Eligible patients will be identified and recruited from their usual haemodialysis session. They will be invited to complete tasks and questionnaires collecting data on sarcopenia, immunosenescence, mood, cognition, disability, and comorbidity. Fifty pre-frail participants with suitable English proficiency will be randomly selected from this cohort to participate in the randomised controlled trial phase of the study. Further stratified randomisation will occur to assign these 50 participants to active or passive groups. The active group will receive a psychologically supported, patient-centred, multi-disciplinary intervention into frailty, in what we believe to be a first within this patient group. The control group will receive usual haemodialysis standard of care. All participants will be followed up using electronic patient records for outcomes to include hospitalisation and mortality. Primary outcomes for this phase of the study will be feasibility and tolerability of the clinical intervention study.

**Discussion:**

The study will collect data on multiple aspects of frailty allowing for a rich dataset for detailed analysis. We believe this will be the first study to explore a psychologically supported, patient-centred intervention in this patient group.

**Trial registration:**

Clinicaltrials.gov, NCT03071107. Registered on 6 March 2017.

**Electronic supplementary material:**

The online version of this article (10.1186/s13063-018-2842-x) contains supplementary material, which is available to authorized users.

## Background

Frailty is a state of low physiological reserve and multi-systemic dysregulation that leaves the individual susceptible to external stressors [1]. Sarcopenia, an age-related, involuntary loss of skeletal muscle mass and strength and/or function is often seen as part of the frailty syndrome [2, 3]. Immunosenescence is another component of frailty, characterised by age-related deterioration in immune function, and is thought to be modifiable through exercise [4]. Frailty is prevalent among patients receiving haemodialysis, ranging between 30% to as high as 78% dependent upon the diagnostic tool used, and is associated with significant adverse outcomes such as falls, hospitalisation, mortality, and loss of functional independence [5–17]. These studies were conducted in North American cohorts, primarily using the Fried Frailty Phenotype, and to date we have no information regarding the prevalence or outcomes associated with frailty in a UK cohort. Dialysis outcomes differ significantly between these populations [18], limiting the generalisability of US data to UK cohorts. Whilst there are a range of validated subjective and objective measurements for frailty, it is unclear which has the most utility to guide clinical practice.

While frailty is not a fixed physiological state, there is currently no evidence to support any intervention targeted to improve frailty within patients receiving haemodialysis. In the general elderly population, there is some evidence of the utility of a multi-disciplinary approach to intervene on frailty. For example, the FIT study utilised a multi-factorial inter-disciplinary intervention which reduced frailty among older people by 14.7% at 12 months [19]; however, there were no other outcomes measured, such as quality of life, mortality, or hospital admissions. The majority of frailty intervention studies performed in both general and dialysis populations do not use validated measures of frailty for either inclusion criteria or outcome measures, but show that functional outcomes (i.e. disability) can be improved with exercise [20–22]. For those patients proceeding to kidney transplantation, frailty at the time of transplantation has been associated with poorer post-transplant outcomes [23–26], although there have not been any published attempts at “prehab” in prospective transplant recipients. Therefore, whether such a strategy would lead to more haemodialysis patients being deemed suitable to benefit from transplantation remains to be seen.

We speculate that patient behaviours can be modified to impact upon frailty. To the best of our knowledge, there have not been any published attempts to intervene upon frailty using behavioural change. Meta-analysis and meta-regression have shown that behavioural change techniques (BCTs) congruent with control theory [27] are significantly more effective than those that are not [28–30]. These include node-link mapping (NLM), which incorporates visual representations of behavioural change, alongside potential aids and barriers to this change with strategies to overcome these [31]. NLM is a technique already successfully used in addiction psychiatry [32]. Social behaviour and network therapy (SBNT) seeks to identify the level of social support for an individual, and aims to include significant supportive others in setting and monitoring goals, and has been successfully used in alcohol abstinence after liver transplantation [33].

It is speculated that long-term changes in behaviour and activity are likely to be required to impact upon frailty and, as such, BCTs may have a role to play in frailty interventions.

## Aims

This study aims to: 1) measure prevalence and outcomes associated with frailty in a large UK haemodialysis cohort; 2) determine the optimum frailty assessment tool for UK patients receiving haemodialysis, as defined by predictive value for mortality/hospitalisation; and 3) conduct a feasibility study exploring a multi-disciplinary clinical intervention to improve frailty status among patients receiving haemodialysis

## Methods/design

This is a prospective, single centre, open-label, cohort multiple randomised controlled trial (cm-RCT) [34] which will be split into two elements. Work package 1 is a cohort study of prevalent haemodialysis patients which aims to identify the prevalence of frailty among this population and outcomes associated with frailty such as mortality, hospitalisation, and quality of life. Patients from work package 1 who are identified as “pre-frail”, as defined by the Frailty Index, will then be invited to participate in work package 2 which will compare the effect of active lifestyle intervention versus current standard of care upon frailty measurements and outcomes. All haemodialysis patients who meet the inclusion/exclusion criteria will be invited to participate in work package 1. As part of the cm-RCT design, eligible patients will be selected for invitation to participate in work package 2 according to a stratified randomisation module. Invited participants will be randomised into active or passive intervention groups. The duration of the clinical intervention in work package 2 will be 6 months, with electronic data-linkage follow-up for 5 years after study participation to allow for continued capture of outcome measures.

### Study setting

Patients will be recruited from a single nephrology centre located in Birmingham, UK. The centre consists of one in-hospital dialysis unit and 10 private-provider satellite units distributed around the region, and provides haemodialysis to 1000 patients. It is planned to recruit at least 602 patients from this population to work package 1 with 50 patients recruited to work package 2. Birmingham and the West Midlands is a diverse, multi-cultural population and, as such, we will aim to recruit proportionate numbers of black, Asian and minority ethnic (BAME) participants to the study to accurately represent the community our hospital serves.

### Inclusion and exclusion criteria

#### Work package 1: inclusion criteria

The inclusion criteria for work package 1 are: 1) aged 18 years and over; 2) received regular haemodialysis of at least 3 months duration; and 3) able to provide informed consent.

#### Work package 1: exclusion criteria

The work package 1 exclusion criterion is having received inpatient care within 4 weeks of recruitment (unless for vascular access).

#### Work package 2: inclusion criteria

The inclusion criteria for work package 2 are: 1) aged 18 years and over; 2) received regular haemodialysis of at least 3 months duration; 3) able to provide informed consent; 4) a participant in work package 1; 5) identified as pre-frail in work package 1; and 6) suitable English proficiency, i.e. an ability to understand a medical consultation in English.

#### Work package 2: exclusion criteria

The exclusion criteria for work package 2 are: 1) deemed unsuitable to complete study (clinician advice); 2) currently enrolled in another clinical intervention trial; 3) receiving emergency inpatient care within 4 weeks of recruitment/assessment (patients admitted for routine procedures, e.g. dialysis access, will be eligible for inclusion); and 4) planned live donor kidney transplant during study period.

### Work package 1

#### Study protocol

Potential participants will be identified through a review of all patients attending haemodialysis sessions, currently available from electronic patient records (EPRs) and from clinicians in charge of each haemodialysis unit. Patient information leaflets (PILs) will be given to the patients in advance of a discussion with the researcher either by post or in person. Individuals will be approached at their usual dialysis unit appointment for discussion and potential recruitment to the study. Potential participants will be given the opportunity to reflect on the information given both verbally and within the PIL. No fixed time is specified for the length or timing of this prior interview as it will be dependent upon the patient’s understanding of their underlying disease and of the research project. It is important that non-English speaking patients are given full opportunity to be informed and recruited to the study, to accurately reflect the demographics of the haemodialysis population. In these instances, we will use any possibility to discuss the study with patients to aid participation and consent. This may include using family, friends, dialysis staff, or the investigator team who may be proficient in the patients preferred language (e.g. Urdu, Punjabi). Written consent will be received from each patient entering the study.

#### Baseline assessment

Prior to the patient being connected to the dialysis machine, the following assessments will take place: 1) timed walk over 4 m; 2) assessment of grip strength with a dynamometer; and 3) Montreal Cognitive Assessment (MoCA)

When the patient is dialysing, the following assessments will be performed: 1) quadriceps ultrasound assessment; 2) questionnaires will be given to participants in either paper or electronic format via a tablet device, according to patient preference, including the 3-level version of the EuroQol five dimensions (EQ-5D-3 L) and the Patient Health Questionnaire (PHQ)-9 (where a PHQ-9 score of 20 or over may indicate depression and a discussion will be held with the patient asking whether they would like a discussion with their nephrologist or a referral to the renal psychology service); and 3) a questionnaire related to each frailty instrument, demographic data, and social history (level of education, employment status, nature of employment, smoking, and alcohol history).

EPRs will be interrogated for data on comorbidities, dialysis parameters, previous transplantation, biochemical data, medication history, and social deprivation score.

The patient’s nephrologist will be invited to complete a Clinical Frail Scale for individual participants within 1 month of the baseline clinical assessment.

#### Data handling

Data collected throughout the study will be entered onto a study-specific database REDCap™ (Research Electronic Data Capture) [35] with secure storage and full oversight from information governance. Any paper records which form part of the source data for the study will be retained securely at site.

Patients will be consented for electronic data capture of clinical parameters (e.g. biochemical data) from University Hospitals Birmingham EPR at 1 month, 3 months, 1 year, 3 years, and 5 years post-recruitment (facilitated through informatics linkage utilising Hospital Patient Registration number). In addition, clinical outcomes (e.g. hospital admissions, medical events, surgical procedures, death, etc.) will be electronically captured from Hospital Episodes Statistics for the same time points (facilitated through informatics data linkage utilising National Health Service (NHS) numbers). This will allow long-term data capture of important clinical outcomes without direct patient involvement.

## Work package 2: plan of investigation

### Study protocol

The FITNESS protocol has been designed and reported in line with SPIRIT guidelines, with an attached SPIRIT flow diagram (see Fig. 2 later) and checklist (Additional file 1) [36].

#### Recruitment and consent

Potential participants will be identified through their previous participation in work package 1. Patients scored as “pre-frail” will randomly be selected to participate in work package 2. Patient information sheets will be given to the patients in advance of a discussion with the researcher either by post or in person. Individuals will be approached at their usual dialysis unit appointment for discussion and potential recruitment to the study. Potential participants will be given the opportunity to reflect on the information given both verbally and within the written information sheet. No fixed time is specified for the length or timing of this prior interview as it will be dependent upon the patient’s understanding of their underlying disease and of the research project. A certain level of English proficiency will be necessary to participate in the intervention, however it is important that non-native English speaking patients are given full opportunity to participate in this study and the same methods will be used as in work package 1 to facilitate this. Written consent will be received from each patient entering the study. A study patient identifier will be allocated and recorded on the study recruitment log.

Work package 1 patients who meet eligibility criteria will be randomly selected for participation in work package 2 using REDCap™ (in random permuted blocks within strata to balance numbers and characteristics). Participants who have subsequently been approached and given consent to participate in work package 2 will be randomised once more using REDCap™ (in random permuted blocks within strata to balance numbers and characteristics) into one of the following lifestyle intervention groups (*n* = 25 for each intervention group for a total of 50 participants). As part of the cm-RCT design, data from pre-frail work package 1 patients who were eligible but not selected for participation in work package 2 will provide a further comparator group for analysis. As an open-label study, neither participants nor researchers will be blinded to group allocation.

#### Active intervention group

This group will receive interventions designed to address frailty and will consist of dietitian referral for dietetic advice and physiotherapist referral for a graded exercise programme. The participants will receive this in addition to their usual haemodialysis standard of care. To reduce potential bias, neither the physiotherapist nor the dietitian will have regular input with haemodialysis patients as part of their job plan. Each patient will have five face-to-face appointments with the dietitian and physiotherapist (lasting 45–60 min) at baseline, week 6, week 12, week 18, and week 24. Brief telephone reviews will be conducted between appointments (approximately2–3 weeks after each face-to-face appointment) to review progress and provide additional support during the active intervention period. Some appointments may be substituted with telephone support if preferred by the patient. Patients will have their diet reviewed by a dietitian and healthy eating advice will be given based upon guidelines issued by the British Dietetic Association [37], which provide recommendations for minimum protein intake in dialysis patients. Patients will be advised to keep food and exercise diaries to monitor compliance with initiated changes and will be followed up by the research team prospectively (using face-to-face appointments and telephone reviews) to monitor progress and reinforce the advice (in addition to routine clinic visits). In addition, a graded exercise programme will be established by the physiotherapist to increase physical activity in line with both the patient’s stated goals at initial assessment and their deficits identified upon frailty scoring.

The dietitian and physiotherapist will be supported by a Senior Lecturer and Consultant in Addiction Psychiatry who has expertise in behavioural change therapy. The dietitian and physiotherapist will be trained with motivational interviewing skills and the following psychological tools will be utilised to support the active intervention.

Cognitive behavioural therapy (CBT) intervention incorporating NLM is simple to train and apply. We will utilise simple goal-setting techniques, combined with relapse prevention strategies, and a simple visual representation system for presenting CBT-type interventions. NLM is more effective than standard consultations for improving the therapeutic alliance, increasing focus on key issues during the session, and improving outcomes. There is also evidence of efficacy in patients with poor reading skills or working in a language other than their first language (important for our patient demographics) [31].

SBNT is an intervention that draws in members of the family, social network, and mutual self-help or peer support groups to help the patient set and achieve goals. It has been developed and tested by colleagues in Birmingham, with an evidence base in the context of liver transplantation [33]. A combination of these two techniques, together with motivational interviewing [38] by the dietitian and physiotherapist after appropriate training, will facilitate supportive psychosocial interventions to change lifestyle behaviour.

#### Passive control group

This group will receive their usual standard of care from their treating dialysis centre, including usual dietitian input. However, there will be no psychosocial intervention or focused exercise and dietary monitoring programme. Follow-up will be at routine clinic visits only, where lifestyle modification advice will be reinforced as per usual clinical practise. Physiotherapy may be offered to these patients as per standard referral guidelines but will not have the psychosocial underpinning of the intervention group.

**Fig. 1:**
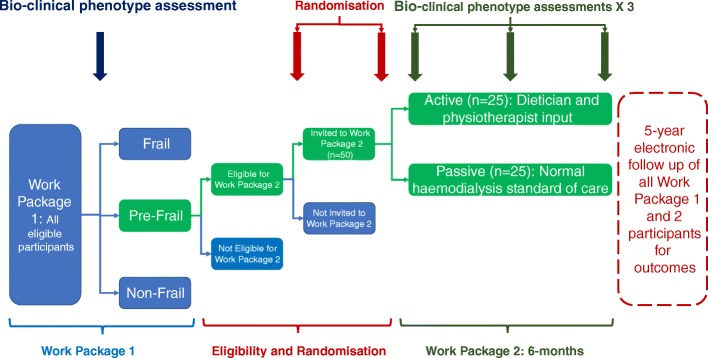
Flowchart of participants in FITNESS work packages 1 and 2. As part of the cm-RCT methodology, all eligible participants will first be randomised for potential invitation to the study (*n* = 50). Potential recruits will then be approached for involvement in work package 2 and, after further valid consent, will then be randomised into active or passive groups

**Fig. 2:**
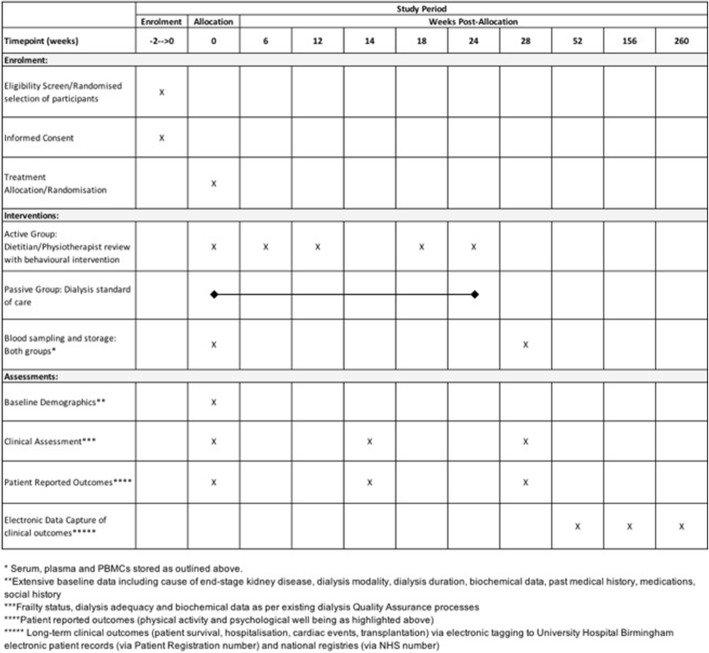
Spirit figure for work package 2

Both groups will undergo a repeat frailty testing during the mid-point (3 months ± 2 weeks) and the end of study intervention (6 months ± 2 weeks) (see Figs. 1 and 2). The aim is to assess if there is any change in frailty status which will provide data to underpin a power calculation for a subsequent large-scale study. Additional pre-specified secondary outcomes will be collected as highlighted below in the protocol. Participants will also be invited to complete a series of questionnaires to ascertain the tolerability and suitability of the intervention trial, and to guide the delivery of the trial on a larger scale.

### Study outcomes

#### Primary endpoint

The primary endpoint is the feasibility and tolerability of the clinical intervention study (recruitment rates, loss to follow-up, and study withdrawal).

#### Secondary endpoints

Secondary endpoints include: 1) refinement of the multi-disciplinary intervention based upon views from participants and stakeholders (from post-study questionnaires); 2) change in frailty status (based on Fried Frailty Phenotype, Clinical Frailty Scale); 3) difference in clinical and biochemical outcomes; 4) quality of life score (EQ-5D-3 L); 5) receipt of kidney transplant; 6) vascular access failure; and 7) long-term clinical outcomes (death, cardiac events, hospitalisation) to University Hospital Birmingham electronic patient records and Hospital Episode Statistics

### Data collection

#### Tissue collection

Blood samples will be taken from the 50 work package 2 participants, alongside 50 work package 1 participants each from the frail and non-frail groups. Plasma, serum, peripheral blood mononuclear cells (PBMCs) and whole blood will be stored for future immunophenotyping.

#### Electronic data capture

Electronic data capture will continue in work package 2 as previously described in work package 1.

#### Data monitoring and risk

There are no specific risks anticipated with this study. However, any study-related adverse events (AEs) will be documented and reported to the steering committee. Should a participant report any thoughts of deliberate self-harm or suicide, or complete a PHQ-9 score of over 20 during the study, a discussion will be held with the patient asking whether they would like a referral to the patient’s nephrologist or psychology services at the trust.

A serious adverse event (SAE) is defined by the Health Research Authority as an untoward occurrence that: 1) results in death; 2) is life-threatening; 3) requires hospitalisation or prolongation of existing hospitalisation; 4) results in persistent or significant disability or incapacity; 5) consists of a congenital abnormality or birth defect; and 6) is otherwise considered medically significant by the investigator.

An SAE occurring to a research participant will be reported to the Research Ethics Committee (REC) where in the opinion of the Chief Investigator the event was: 1) related (it resulted from the administration of any of the research procedures); and unexpected (the type of event is not listed in the protocol as an expected occurrence).

Any SAE will be reported to the REC within 15 days of the Chief Investigator becoming aware of the event. All other SAEs will need reporting as AEs on the case report form.

Exceptions to expedited reporting include hospitalisation for: 1) treatment which was elective or pre-planned, or for a pre-existing condition not associated with any deterioration in condition, e.g. pre-planned hip replacement operation which does not lead to further complications; and 2) treatment on an emergency, outpatient basis for an event not fulfilling any of the definitions of serious as given above and not resulting in hospital admission.

Patients will be asked if any adverse events have occurred when they attend for any trial-related procedure.

#### Participant withdrawal

In the event of patients wishing to discontinue in the trial, the participant would be withdrawn from the study with the assurance that their usual care will continue unaffected. Identifiable data or tissue already collected with consent would be retained and used in the study. No further data or tissue would be collected, or any other research procedures carried out on or in relation to the participant. If the patient consented to have long-term electronic tracking of their outcomes, this would be continued.

#### Statistics

The principle parameters being examined in work package 1 are hospitalisation, mortality, and their association with frailty. Based upon US data [6], we have assumed an adjusted risk ratio of 2.24 for 1-year mortality and 1.56 for 1-year mortality and/or hospitalisation for frail versus non-frail patients receiving haemodialysis. We have assumed a baseline (non-frail) risk of 5% for 1-year mortality and a 40% risk of 1-year mortality/hospitalisation, powered to 0.8 and with a confidence interval of 0.95. We therefore calculate a sample size of 602 to be adequately powered to demonstrate a difference in 1-year mortality or 150 patients to be powered for 1-year mortality/hospitalisation.

Work package 2 is designed as a feasibility study so no specific power calculations have been made. However, one of the secondary outcomes is to use the data regarding effect sizes to determine the sample required for a larger multi-centre study based upon the feasibility of work package 2.

Statistical analysis will be performed using standard software (SPSS Version 25, Mac version, Chicago, USA). Normality of data will be assessed using the Kolmogorov-Smirnov tests. Paired sample *t* test and Wilcoxon signed rank test, for parametric and non-parametric data respectively, will be used to compare the means of two variables from a single group. Comparison of data between groups will be made using unpaired student *t* tests and the Mann-Whitney test for parametric and non-parametric data, respectively. Categorical data will be analysed using Pearson’s or Spearman’s test as appropriate. A *p* value < 0.05 is considered significant in the statistical analysis.

## Discussion

Frailty has been linked with adverse outcomes in North American haemodialysis cohorts [5–12, 17, 39, 40], but these data are not transferrable to UK patients since US dialysis populations have adverse outcomes compared with UK populations [18]. There is no agreement over the best frailty tool to assess patients, both within the general population and within haemodialysis cohorts. The Fried Frailty Phenotype [41] and Rockwood Frailty Index [42] are the most commonly cited instruments in geriatric and renal-specific literature, with the Clinical Frailty Scale also featuring in haemodialysis cohorts [12]. The Edmonton Frail Scale is recommended by the British Geriatric Society, but has yet to be validated in patients receiving haemodialysis [43]. We expect each of these instruments to show association with negative outcomes, but it is not yet known which of these provides the best predictive value in patients receiving haemodialysis. The cohort analysis embedded within this study will therefore seek to address this by comparing frailty instruments for their association with negative outcomes, whilst also addressing renal-specific outcomes such as transplantation or dialysis access failure. These data will allow renal practitioners to better understand which frailty tool is optimal for their population.

Multi-disciplinary intervention into frailty has shown promise [19], although a systematic review and meta-analysis was limited by the lack of validated measures of frailty as inclusion criteria and outcome measures in the published studies to date [22]. To our knowledge, there are no published data regarding frailty interventions within the UK haemodialysis population, although a US study showed self-reported disability could be reduced by such an intervention [20].

There is an urgent need for the development of interventions capable of enacting long-term change within patient groups. Our experience in Birmingham suggests that BCTs can modify behaviour with the potential to change clinical outcomes [33, 44]. Therefore, a patient-centred, frailty-specific BCT intervention may be beneficial for frail patients receiving haemodialysis and may improve clinical and patient-reported outcomes.

Work package 2 is an unblinded, randomised controlled study using a psychologically supported multi-disciplinary intervention into frailty compared with a control group which receives standard care. This will be used as a basis for exploring whether a large scale, multi-centre trial is feasible and warranted, while also providing data upon effect sizes to allow subsequent power calculation for such a study. We chose the “pre-frail” group as the focus of this intervention, firstly because they arguably have the most to gain—not progressing to frailty—and, secondly, because pre-frailty represents a much less heterogeneous group than frailty, the latter of which would include essentially functionally independent individuals with a few accumulated deficits along with bed-bound individuals and those approaching end-of-life. The pre-frail group therefore have an easily definable set of characteristics and have the ability to participate in, and benefit from, the proposed interventions.

One of the principle strengths of the study design is the breadth of data being collected. Researchers will collect data on multiple aspects of frailty from consenting patients to include aspects of sarcopenia (muscle mass, quality, and function), immunosenescence, functional status/disability, mood, cognitive status, and medical history. This rich dataset will allow detailed analysis of the heterogeneity or otherwise of frailty within the cohort. As work package 1 is a single time-point analysis with electronic follow-up thereafter, dropout should be minimal and large numbers of patients could be recruited to allow such detailed analysis to have real meaning to clinicians. Another strength lies in the cm-RCT design, whereby stratified random sampling will identify trial participants from consenting eligible cohort study participants. This approach has several benefits, including amelioration of potential attrition bias in the control group, long-term data follow-up for a large comparator group, and increased power for the study [34].

A weakness of the study is the single time-point assessment in work package 1, as frailty is known to change over time [45–47]. Nevertheless, objective frailty measurements still hold prognostic value [48].

This cohort randomised controlled trial will assess the prevalence and associations of frailty using validated measurement tools and test the utility of these instruments in predicting adverse outcomes. The study will also assess the feasibility of conducting a randomised controlled trial using an intervention designed to improve frailty.

## Additional file

Additional file 1: SPIRIT 2013 checklist: recommended items to address in a clinical trial protocol and related documents. (DOC 121 kb)

## Abbreviations

AE: Adverse event
BAME: Black, Asian and minority ethnic
BCT: Behavioural change technique
CBT: Cognitive behavioural therapy
cm-RCT: Cohort multiple randomised controlled trial
EPR: Electronic patient record
MoCA: Montreal Cognitive Assessment
NHS: National Health Service
NLM: Node-link mapping
PHQ: Patient Health Questionnaire
PIL: Patient information leaflet
REC: Research Ethics Committee
REDCap: Research Electronic Data Capture
SAE: Serious adverse event
SBNT: Social behaviour and network therapy
